# Postoperative oxaliplatin-based hyperthermic intraperitoneal chemotherapy: an effective and safe palliative treatment option for colorectal cancer with peritoneal metastasis

**DOI:** 10.1186/s12957-021-02320-4

**Published:** 2021-07-06

**Authors:** Tuanhe Sun, Kang Li, Gang Xu, Kun Zhu, Qiong Wang, Chengxue Dang, Dawei Yuan

**Affiliations:** Department of Surgical Oncology, First Affiliated Hospital of Xi’an Jiaotong University, Yanta west street No.277, Xi’an, 710061 Shaanxi China

**Keywords:** Hyperthermic intraperitoneal chemotherapy, Colorectal cancer, Peritoneal metastasis, Ascites, Cytoreductive surgery

## Abstract

**Background:**

The prognosis of patients with colorectal cancer and peritoneal metastasis (CRC-PM) after incomplete cytoreductive surgery (CRS) or palliative surgery is poor. Novel and effective therapies are urgently needed. This study aimed to assess the effects of palliative postoperative hyperthermic intraperitoneal chemotherapy (HIPEC) in patients with CRC-PM.

**Methods:**

This retrospective study included patients with CRC-PM at the First Affiliated Hospital of Xi’an Jiaotong University in 05/2014–05/2019. Observation indicators included overall survival (OS), ascites-free survival, peritoneal cancer index (PCI), and completeness of cytoreduction (CC). Kaplan-Meier survival curves and multivariable Cox regression models were used to determine the factors associated with OS and ascites-free survival. The ascites-specific quality of life (QoL) was measured using the Functional Assessment of Chronic Illness Therapy-Ascites Index (FACIT-AI).

**Results:**

Eighty-two patients were included, including 37 and 45 in the HIPEC and non-HIPEC groups, respectively. Mean OS was 10.3±3.7 (95% CI 9.5–11.2) months. Multivariable Cox proportional hazard regression suggested that PCI (HR=6.086, 95% CI 3.187–11.620, *P* < 0.0001) was independently associated with OS. The degree of ascites (HR=2.059, 95% CI 1.412–3.005, *P* < 0.0001), PCI (HR=6.504, 95% CI 2.844–14.875, *P* < 0.0001), and HIPEC (HR=0.328, 95% CI 0.191–0.562, *P* < 0.0001) were independently associated with ascites-free survival. In patients with survival >6 months, postoperative ascites-specific QoL was significantly improved after HIPEC compared with the non-HIPEC group (*P* < 0.001). Oxaliplatin-based HIPEC significantly increased the rates of neutropenia and peripheral neurotoxicity (both *P* < 0.05).

**Conclusion:**

These data indicate that postoperative oxaliplatin-based HIPEC might help increase ascites-free survival in CRC-PM patients after incomplete CRS or palliative surgery, with improved QoL after 6 months of follow-up.

## Background

Colorectal cancer (CRC) is a malignant neoplasm of the colon or rectum and represents the third most common cancer worldwide [1–3]. The incidence and mortality of CRC are steadily increasing in developing countries, especially in China, where CRC incidence has increased at an average annual rate of 3–4% over the past three decades [4]. The peritoneum is the second most common site of metastasis in patients with CRC; indeed, about 5–10% of CRC patients have peritoneal metastasis at the time of diagnosis, with the peritoneum being the only distant metastatic site for 10–15% of these patients [5].

CRC with peritoneal metastasis (CRC-PM) is commonly treated by systemic chemotherapy [6, 7], but the patient prognosis is very poor, and survival is significantly reduced compared with patients with liver or lung metastasis [8]. With recent advances in the understanding of tumor biology, peritoneal metastasis is gradually considered a regional lesion rather than systemically disseminated metastases [9, 10]. Cytoreductive surgery (CRS) combined with hyperthermic intraperitoneal chemotherapy (HIPEC) has become a new treatment option for peritoneal metastasis, with good efficacy in a variety of malignant tumors, including ovarian cancer [11], gastric cancer [12], pseudomyxoma peritonei [13], and CRC [14]. This combination therapy can minimize the tumor burden using CRS and kill free tumor cells and micrometastases using HIPEC [15–17].

In addition to the traditional role of intraperitoneal chemotherapy, HIPEC has multiple advantages, including the direct killing effect of hyperthermia on cancer cells, the chemotherapy-sensitizing effect of hyperthermia, and the mechanical scouring effect of fluid circulation in the peritoneal cavity [17, 18]. Previous clinical studies showed that HIPEC combined with complete CRS confers significant survival benefits in patients with CRC-PM [14, 19], but recent studies have reported controversial results that complete CRS, rather than HIPEC, is the key for improving patient survival [20, 21]. Indeed, recent trials reported no benefits in terms of survival [20, 22, 23]. In addition, a study suggested that one patient over seven who undergo CRS achieves long-term survival [24]. In addition, due to the extent of the disease and the surgeon’s experience and skills, not all patients can undergo complete/satisfactory CRS. Therefore, whether HIPEC should be administered in patients after palliative surgery or incomplete CRS (completeness of cytoreduction [CC] score of 2 or 3) remains unknown.

Therefore, this retrospective study aimed to assess the effects of postoperative oxaliplatin-based HIPEC in patients with CRC-PM after incomplete CRS or palliative surgery.

## Methods

### Study design and patients

This retrospective study included patients with CRC-PM who underwent palliative surgery (enterostomy, colostomy, or bypass) or incomplete CRS at the First Affiliated Hospital of Xi’an Jiaotong University between May 2014 and May 2019.

The inclusion criteria were (1) >18 years of age and (2) peritoneal metastases diagnosed at the same time as the primary CRC (CRC above the peritoneal reflection). The diagnosis of CRC was based on pathological biopsy during endoscopy or intraoperative exploration [25]. The diagnosis of peritoneal metastasis was based on preoperative imaging examination, cytological examination of ascites, or intraoperative exploration [26]. The exclusion criteria were (1) systemic chemotherapy or targeted drug therapy within 3 months before the operation; (2) CC score of 0 or 1, indicating complete CRS; (3) concomitant severe heart, lung, liver, kidney, or other dysfunction, or severe abdominal adhesion; or (4) Karnofsky Performance Score (KPS) <60.

The study was approved by the ethics committee of the First Affiliated Hospital of Xi’an Jiaotong University. The need for individual consent was waived by the committee because of the retrospective nature of the study.

### CRS

All patients are evaluated with KPS before surgery. After general anesthesia, the patient’s abdominal cavity was entered through a mid-abdominal incision to explore and evaluate the peritoneal cancer index (PCI) [27]. According to the current CRS standard procedure [28], primary lesions, peritoneal metastases, and the involved organs or tissues were removed according to the region, and lymph nodes were dissected. Each tumor was reduced to the maximum extent, and the CC score was evaluated before abdominal closure.

The distribution of peritoneal deposits was assessed using the PCI system [29]. The total scores ranged from 0 to 39. The completeness of CRS was evaluated by the CC score: CC-0 resection, no tumor detected after complete resection; CC-1 resection, residual tumor nodules <2.5 mm in both diameters; CC-2 resection, residual tumors between 2.5 mm and 2.5 cm in diameter; CC-3 resection, residual tumors >2.5 cm in diameter. In this study, an incomplete CRS was defined as a CC score of 2 or 3. In our center, the patients undergoing CRS have a preoperative KPS ≥70, and patients undergoing palliative surgery (including incomplete CRS (CC ≥2)) might have a lower preoperative KPS (KPS=60). Therefore, the patients were divided into KPS 60 vs. ≥70. According to the recommendations for the treatment of peritoneal cancer from CRC with CRS and HIPEC in a multicenter retrospective study, PCI of 20 or lower had to be justified [14]. When the PCI is greater than 20, the 5-year survival rate is less than 10%, which indicates that extensive disease becomes a relative contraindication for this combined treatment [14]. Therefore, the patients were grouped according to a PCI score of 20.

### HIPEC

Postoperative HIPEC was proposed to all patients with CRC-PM without CRS (i.e., with palliative surgery only) or with incomplete CRS. Before closing the abdomen, four drainage tubes were placed on top of the liver, in the splenic fossa, and on both sides of the pelvic cavity. HIPEC was the first postsurgical treatment to be administered and was started on the first day after the operation. Using the hyperthermic perfusion intraperitoneal treatment system (BR-TRG-II, Baorui Medical Technology Co., Ltd., China), oxaliplatin (ELOXATIN®, CENEXI-Laboratories THISSEN S.A., Belgium or Qisha®, Qilu Pharmaceutical Co., Ltd., China) was mixed with 3000–4500 ml of 5% glucose, heated at a constant temperature of 42 to 43°C, and continuously and circularly perfused for about 60 min. The temperature of the perfusate was measured in the outflow (≥40°C), and the patients’ armpit temperature was measured every 20 min. Non-steroidal anti-inflammatory analgesics, allergy prevention, and other drugs were given during the treatment to reduce the patient’s discomfort and adverse reactions. During HIPEC, the vital signs and blood glucose levels of the patients were monitored, and symptomatic treatments were provided, including pain relief, blood sugar control, allergic reaction prevention, and anti-emesis. The perfusion fluid was slowly drawn at about 4–6 h after HIPEC completion. The total amount of oxaliplatin was 360 mg/m^2^ and was administered 3 or 4 times within 7 days after the operation.

### Systemic chemotherapy and targeted therapy

According to the specific patient conditions, systemic chemotherapy (based on fluorouracil: mFOLFOX6, CapeOx, or FOLFIRI) was administered within 2–8 weeks after CRS. During the treatments, the general condition, blood routine parameters, liver and kidney functions, electrocardiograms, and imaging examinations were routinely monitored. Twenty-one patients were treated with a targeted drug in addition to chemotherapy (i.e., bevacizumab or cetuximab).

### Follow-up, observation indicators, and related definitions

The preplanned observation period was 24 months, but no patients were alive by then. All follow-up data were from the patient charts. In patients with CRC-PM, there is no fixed follow-up plan, and follow-up is individualized. The observation indicators included overall survival (OS, defined as the time from the first diagnosis of CRC-PM to death), ascites-free survival, PCI score, and CC score. According to the consensus by the International Ascites Club, the degree of ascites was graded as [30]: grade 1 or small ascites, mild ascites only detectable by ultrasound examination; grade 2 or moderate ascites, moderate symmetrical distension of the abdomen; grade 3 ascites or large ascites, marked abdominal distension. Ascites-free survival was defined as the time between the operation and the occurrence of moderate ascites or death.

### Quality of life

The patient’s QoL was measured routinely using the Functional Assessment of Chronic Illness Therapy-Ascites Index (FACIT-AI), previously developed for malignant ascites by selecting relevant questions from the FACIT library [31]. The assessed symptoms include anorexia, insomnia, reduced mobility, dyspnea, nausea, vomiting, abdominal pain, abdominal distension, fatigue, early satiety, urinary frequency, constipation, and emotional distress. These 13 symptoms are scored on a 5-point Likert scale from “not at all” to “very much” (range 0–4). Higher scores indicate better QoL. QoL data were collected at baseline (preoperatively within 2 weeks before the day of surgery) and at 1, 3, 6, 9, and 12 months after surgery. A trained investigator contacted all the participants in person or via telephone.

### Statistical analysis

Continuous data were presented as mean ± standard deviation (SD) and analyzed using Student’s t-test. Categorical data were presented as numbers and percentages (n, %) and analyzed by the chi-square test or Fisher’s exact test, as appropriate. Survival curves were obtained by the Kaplan-Meier method and compared using the log-rank test. Independent factors associated with patient survival were identified by Cox proportional hazards regression analysis, determining hazard ratios (HRs) and 95% confidence intervals (CIs). The significance level was set to *P* < 0.05. Data analysis was performed with SPSS 20.0 (IBM, Armonk, NY, USA) and Graph Pad Prism 5 (GraphPad Software Inc., San Diego, CA, USA).

## Results

### Demographic data

This study included 82 patients with CRC-PM. All patients underwent palliative surgery or incomplete CRS (CC score of 2 or 3). Among them, 37 patients received HIPEC and systemic chemotherapy after CRS, while 45 patients received systemic chemotherapy only. There were 48 (58.5%) males. The mean patient age at surgery was 62.5±7.5 years (range, 39–75 years). The detailed demographic and clinical data are shown in Table 1. There were no differences between the two groups in sex, age, KPS, PCI, degree of ascites, distant metastases, primary CRC sites, and the use of targeted drugs (all *P* > 0.05).

**Table 1:** Baseline patient data (*n* = 82)

Clinical variable	HIPEC (*n* = 37)	Non-HIPEC (*n* = 45)	P
Sex, n (%)			
Male	21 (56.8)	27 (60.0)	0.767
Female	16 (43.2)	18 (40.0)	
Age (years, mean±SD)	63.6±7.0	61.6±7.8	0.283
KPS, n (%)			
≥70	33 (91.9)	39 (86.7)	0.724
60	3 (8.1)	6 (13.3)	
PCI, (scores, mean±SD)	12.86±6.19	13.09±7.45	0.884
PCI, n (%)			
<20	29 (78.4)	34 (75.6)	0.763
≥20	8 (21.6)	11 (24.4)	
Degree of ascites, n (%)			
Small or no	22 (59.5)	29 (64.4)	0.751
Moderate	10 (27.0)	9 (20.0)	
Large	5 (13.5)	7 (15.6)	
Distant metastasis, n (%)			
Yes	7 (18.9)	9 (20.0)	0.902
No	30 (81.1)	36 (80.0)	
Site of primary, n (%)			
Right colon	9 (24.3)	7 (15.6)	0.713
Left colon	17 (45.9)	24 (53.3)	
Sigmoid	6 (16.2)	6 (13.3)	
Rectum	5 (13.5)	8 (17.8)	
Targeted drug, n (%)			
Yes	12 (32.4)	9 (20.0)	0.199
No	25 (67.6)	36 (80.0)	

*HIPEC* hyperthermic intraperitoneal chemotherapy, *KPS* Karnofsky performance scale, *PCI* peritoneal cancer index

### Associations of HIPEC and other indicators with OS

**Fig. 1:**
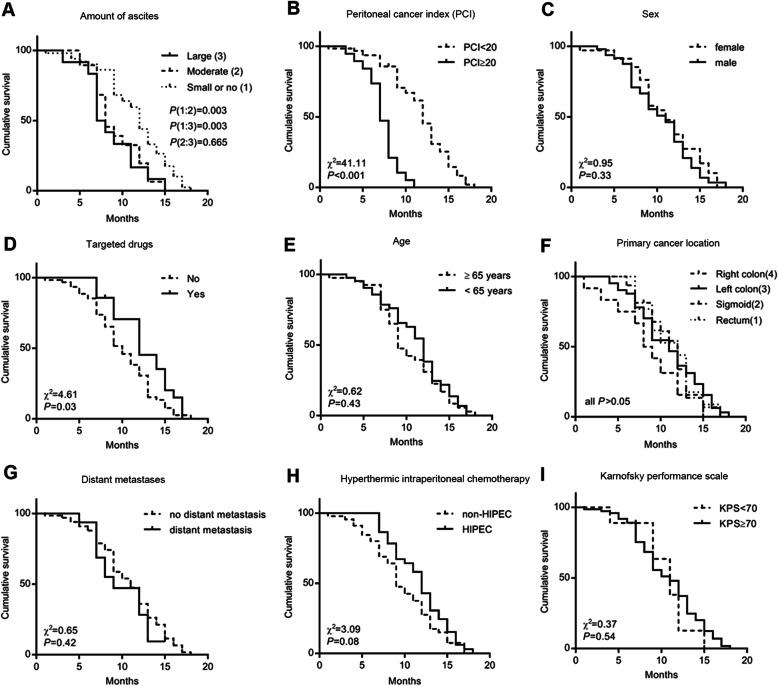
Kaplan-Meier curves for overall survival analysis in patients with colorectal and peritoneal metastasis according to different factors

Mean OS was 10.3±3.7 (95% CI 9.5–11.2) months. The univariable analyses suggest that the degree of ascites, PCI, and treatment with a targeted drug was associated with OS (all *P* < 0.05). Mean OS was prolonged in patients treated with HIPEC (11.4±3.2 months, 95% CI 7.7–9.9) compared with the non-HIPEC group (9.5±3.8 months, 95% CI 8.3–10.6), but the difference was not statistically significant (P=0.08) (Fig. 1H).

The significant factors in the univariable analyses were included in the Cox multivariable analysis. The results suggest that only PCI (HR=6.086, 95% CI 3.187–11.620, *P* < 0.0001) was independently associated with OS (Table 2).

**Table 2:** Cox proportional hazards regression model for analyzing prognostic factors associated with overall survival

Clinical variable	Univariable			Multivariable		
	HR	95.0% CI	P	HR	95.0% CI	P
Sex	1.234	0.772–1.974	0.380			
Age	1.179	0.746–1.861	0.480			
KPS	0.810	0.386–1.700	0.577			
PCI	6.086	3.187–11.620	<0.0001	6.086	3.187–11.620	<0.0001
Degree of ascites	1.626	1.203–2.198	0.002			
Distant metastasis	1.270	0.677–2.382	0.457			
Site of primary	1.034	0.818–1.307	0.779			
Targeted drug	0.599	0.353–1.015	0.057			
HIPEC	0.692	0.436–1.096	0.117			

*HR* hazard ratio, *CI* confidence interval, *KPS* Karnofsky performance scale, *PCI* peritoneal cancer index, *HIPEC* hyperthermic intraperitoneal chemotherapy

### Association of HIPEC and other parameters with ascites-free survival

**Fig. 2:**
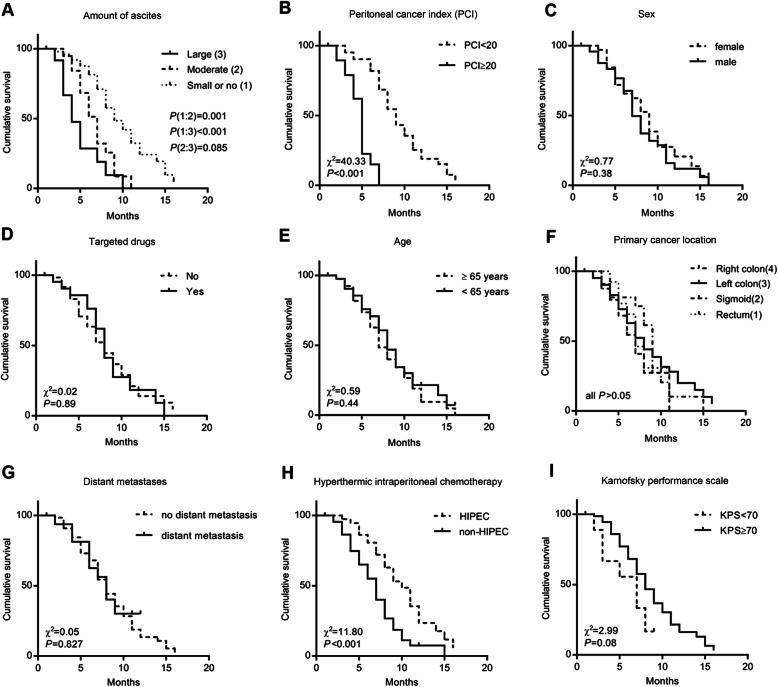
Kaplan-Meier curves for ascites-free survival analysis in patients with colorectal and peritoneal metastasis according to different factors

The mean ascites-free survival was 7.4±3.3 (95% CI 6.7–8.2) months. Univariable analyses suggest that the degree of ascites, PCI, and HIPEC was associated with ascites-free survival (*P* < 0.05). The mean ascites-free survival was significantly prolonged in patients treated with HIPEC (8.8±3.3 months, 95% CI 7.7–79.9) compared with the non-HIPEC group (6.3±2.9 months, 95% CI 5.5–7.2), as shown in Fig. 2 (*P* < 0.001).

Significant factors (*P* < 0.10) in the univariable analyses were included in the Cox multivariable analysis. The results suggest that the degree of ascites (HR=2.059, 95% CI 1.412–3.005, *P* < 0.0001), PCI (HR=6.504, 95% CI 2.844–14.875, *P* < 0.0001), and HIPEC (HR=0.328, 95% CI 0.191–0.562, *P* < 0.0001) was independently associated with ascites-free survival (Table 3).

**Table 3:** Cox proportional hazards regression model for analyzing prognostic factors for ascites-free survival

Clinical variable	Univariable			Multivariable		
	HR	95.0% CI	P	HR	95.0% CI	P
Sex	1.239	0.741–2.070	0.414			
Age	1.200	0.728–1.976	0.475			
KPS	0.518	0.231–1.159	0.109			
PCI	7.708	3.645–16.296	<0.0001	6.504	2.844–14.875	<0.0001
Degree of ascites	2.223	1.581–3.125	<0.0001	2.059	1.412–3.005	<0.0001
Distant metastasis	0.931	0.470–1.844	0.838			
Site of primary	1.089	0.836–1.419	0.526			
Targeted drug	1.036	0.590–1.819	0.901			
HIPEC	0.433	0.256–0.732	0.002	0.328	0.191–0.562	<0.0001

*HR* hazard ratio, *CI* confidence interval, *KPS* Karnofsky performance scale, *PCI* peritoneal cancer index, *HIPEC* hyperthermic intraperitoneal chemotherapy

### Short-term adverse events

The most common adverse events occurring within 30 days after CRS are shown in Table 4. Oxaliplatin-based HIPEC significantly increased the occurrence rates of neutropenia (32.4% vs. 8.9%, P=0.007) and peripheral neurotoxicity (24.3% vs. 4.4%, P=0.019), which was probably related to oxaliplatin use. According to the grading standard of the National Cancer Institute’s Common Terminology Criteria for Adverse Events (NCI-CTCAE, Version 5.0) [32], there were four grade 3 neutropenia, one grade 3 thrombocytopenia, and two grade 3 diarrhea cases in the HIPEC group; in the non-HIPEC group, there were one grade 3 nausea and one grade 3 diarrhea cases. In addition, there were two and one anastomotic fistula cases in the HIPEC and control groups, respectively, as well as one anastomotic bleeding and one severe lung infection case in the HIPEC group. After symptomatic treatment, the adverse events were alleviated or improved by the time of hospital discharge. No operation or HIPEC-related death occurred in this study.

**Table 4:** Adverse reactions in both groups of patients (*n* = 82)

Adverse event (<30 days)	HIPEC (*n* = 37)	Non-HIPEC (*n* = 45)	P
Nausea/vomiting, n (%)	13 (35.1)	15 (33.3)	0.864
Diarrhea, n (%)	17 (45.9)	22 (48.9)	0.791
Fever, n (%)	12 (32.4)	10 (22.2)	0.299
Neutrophil count decreased, n (%)	12 (32.4)	4 (8.9)	0.007
Aminotransferase increased, n (%)	6 (16.2)	3 (6.7)	0.287
Creatinine increased, n (%)	9 (24.3)	6 (13.3)	0.200
Peripheral neurotoxicity, n (%)	9 (24.3)	2 (4.4)	0.019

### Ascites-specific QoL

**Fig. 3:**
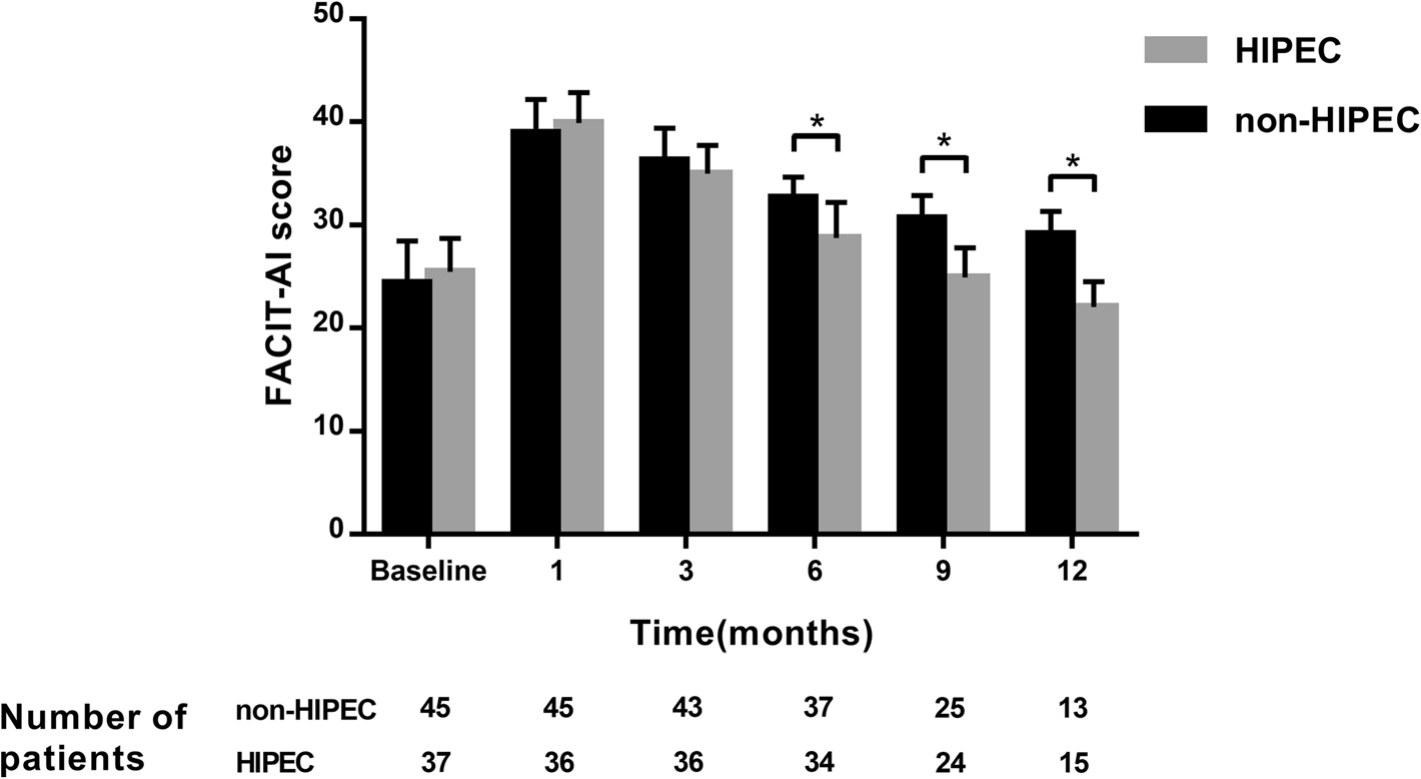
Ascites-specific QoL measured by the FACIT-AI score. *P < 0.001. Data are mean±SD. The higher the FACIT-AI, the more pronounced the improvement

Mean baseline FACIT-AI values were 25.5±3.3 and 24.4±4.1 in the HIPEC and non-HIPEC groups, respectively. Significant improvement in QoL was observed as early as 1 month after surgery, with scores of 39.9±2.9 and 38.9±3.2 in the HIPEC and non-HIPEC groups, respectively. In this study, postoperative ascites-specific QoL in all patients continued to decline over time. When patient survival exceeded 6 months (6, 9, and 12 months), ascites-specific QoL was significantly better in patients treated with HIPEC compared with the non-HIPEC (*P* < 0.001; Fig. 3).

## Discussion

CRC-PM has few effective treatment options, and the prognosis is poor [5, 8], but CRS with postoperative oxaliplatin-based HIPEC might change this situation. CRS is the main factor that affects the prognosis of those patients, but it is extremely difficult to perform a complete high-quality CRS. The prognosis is not only dependent on the technical level of the surgeon but also on the condition of the patients themselves and the number, size, and extent of the lesions in the peritoneum. Therefore, when a satisfactory CRS cannot be completed, is it necessary for these patients to undergo HIPEC? Although HIPEC did not significantly extend the OS, it significantly reduced the occurrence of malignant ascites and improved the QoL. Therefore, it could be concluded that HIPEC might be an effective palliative treatment for these patients. The selection of the HIPEC chemotherapeutic agent and postoperative chemotherapy regimen can also influence the results, and future studies should look for the most effective combinations.

With traditional systemic chemotherapy, the prognosis of CRC-PM patients is very poor [6, 7, 33], while CRS+HIPEC therapy might change that. As early as 2014, CRS+HIPEC was recommended as the standard of care for selected patients with CRC-PM at the Ninth International Congress on Peritoneal Surface Malignancies held in Amsterdam (The Netherlands) [34]. It is estimated that more than 3800 patients with CRC-PM (synchronous and metachronous) were treated with CRS and HIPEC in 430 centers around the world in 2018 [34].

There are two main types of HIPEC applications. The first involves the prophylactic treatment, which is suitable for CRC patients without peritoneal metastases with high-risk factors, e.g., T4 stage and tumor rupture or perforation. In this case, HIPEC is used to prevent the occurrence of peritoneal metastases after radical resection. The other type is therapeutic for patients with CRC who already have peritoneal metastases, and CRS+HIPEC can be used for treatment [14].

In terms of prevention, the COLOPEC study showed that prophylactic HIPEC after radical resection of colon cancer with a high risk of peritoneal metastases (T4 stage or tumor perforation) does not improve peritoneal metastases-free survival (PMFS) at 18 months, with no significant differences in disease-free survival (DFS) and OS [22]. In terms of treatment, the PRODIGE 7 study published in 2018 showed that in patients with CRC-PM administered CRS, additional HIPEC does not prolong DFS and OS and increases complications [20]. However, additional HIPEC could provide survival benefits in patients with a PCI of 11 to 15 [20]. Therefore, some authors believe that completeness of CRS could be the key to prolonging survival in patients and that HIPEC provides no significant benefits [21]. This view corroborates Glehen et al. [23], who retrospectively analyzed 506 patients with CRC-PM, and found that those who had complete CRS (CC score of 0 or 1) had an average survival time of 32.4 months, versus 8.4 months in individuals with a CC score 2 or 3.

Nevertheless, the implementation of complete CRS is challenging for both the surgical team and patients. This operation has a very long learning curve and a high requirement for patient fitness [35, 36]. In addition, studies showed that among patients with CRC-PM administered complete CRS and intraperitoneal chemotherapy, there are significant statistical differences in prognosis among those with PCI <10, PCT 10–20, and PCI>20 (5-year OS of 53%, 23%, and 12%, respectively, *P* < 0.001). Therefore, the prognosis of patients with PCI >20 is considered to be very poor, making them unsuitable for CRS [37]. On the other hand, using current imaging methods, such as CT and PET/CT scans, the sensitivity for detecting peritoneal metastases is only 72.4–87% [38, 39], which affects the surgeon’s accurate preoperative assessment of PCI and reduces the odds of patients receiving complete CRS. The low sensitivity of CT and PET-CT for CRC-PM might even lead to patients undergoing CRS while they could not benefit from it. Therefore, this study examined whether HIPEC could benefit patients after incomplete CRS.

We examined patients with CRC-PM who had incomplete CRS (CC score of 2 or 3) or palliative surgery with or without postoperative HIPEC. The results suggested that HIPEC had a significant advantage in controlling malignant ascites in those patients. A study showed that HIPEC combined with systemic chemotherapy also achieve ascites control, with few adverse events [40]. In addition, except for two side effects associated with oxaliplatin, i.e., neutropenia and neurotoxicity, HIPEC did not significantly increase the incidence of adverse events within 30 days after the operation. All adverse events were relieved or improved at discharge after symptomatic treatment. Other chemotherapy drugs could be explored for use in HIPEC, which could decrease neutropenia and neurotoxicity. No operation or HIPEC-related death occurred in all patients. Nevertheless, this study also suggested that HIPEC did not significantly benefit patients in terms of OS, corroborating previous clinical findings [20]. A recent study highlighted differences in OS after CRS and HIPEC in patients with synchronous vs. metachronous metastasis [41]. In the present study, all patients had CRC-PM at diagnosis. HIPEC can control tumor progression in the abdominal cavity and ascites formation, improve the quality of life of patients, and may allow patients to receive more aggressive anti-tumor regimens, thereby increasing OS. Still, this study does show that the OS has not improved. On the one hand, it might be because these advanced patients are indeed overloaded with tumors, and local treatment in the abdominal cavity cannot control the cancer burden throughout the body. On the other hand, the number of patients was relatively small.

CRC-PM is often accompanied by obvious symptoms, including malignant ascites and intestinal obstruction, different from liver and lung metastases, so active treatment might significantly improve the QoL of the patients. Still, no generally accepted evidence-based guidelines are available for the efficient treatment of malignant ascites [42]. According to the above results, postoperative treatment with appropriate HIPEC could still achieve controlling ascites in patients with CRC-PM not receiving complete CRS for various reasons, thus improving their postoperative ascites-specific QoL.

This study suggested that targeted drugs did not significantly increase ascites control and improve OS in patients with CRC-PM. Due to economic status, the personal wishes of the patients, or other reasons, few patients used targeted drugs in this study, which might affect the results. Meanwhile, the important role of targeted drugs in metastatic CRC cannot be ignored [43]. The associations of targeted therapy’s efficacy, CRS, and HIPEC still need to be defined in future large studies.

The limitations of the present study should be mentioned. First, it was a retrospective analysis with inherent shortcomings. Secondly, the sample size was relatively limited. Thirdly, the patients had complex treatment plans, precluding a detailed stratified analysis, e.g., subgroup analyses based on specific operation methods and postoperative chemotherapy regimens. Fourthly, the patient’s QoL was measured using the FACIT-AI, which was not developed for malignant ascites. Finally, probably only patients with lower initial PCI and a more radical cytoreduction might have been given HIPEC. More comprehensive and detailed evaluation methods would better evaluate patients’ quality of life, and large randomized control studies are needed to verify the present results.

## Conclusion

This study suggests that postoperative oxaliplatin-based HIPEC might help increase ascites-free survival in CRC-PM patients after incomplete CRS or palliative surgery, with improved QoL after 6 months of follow-up. Therefore, postoperative HIPEC might be considered an alternative palliative treatment option for CRC-PM. However, no OS benefit was conferred by this treatment. These results have to be confirmed using a randomized controlled trial.

## Data Availability

The datasets used and/or analyzed during the current study are available from the corresponding author on reasonable request.

## Abbreviations

CRC-PM: Colorectal cancer with peritoneal metastasis
CRS: Cytoreductive surgery
HIPEC: Hyperthermic intraperitoneal chemotherapy
OS: Overall survival
PCI: Peritoneal cancer index
CRC: Colorectal cancer
CC: Completeness of cytoreduction
QoL: Quality of life
FACIT-AI: Functional Assessment of Chronic Illness Therapy-Ascites Index
